# The Influence of the COVID-19 Epidemic on Prevention and Vaccination Behaviors Among Chinese Children and Adolescents: Cross-sectional Online Survey Study

**DOI:** 10.2196/26372

**Published:** 2021-05-26

**Authors:** Zhiyuan Hou, Suhang Song, Fanxing Du, Lu Shi, Donglan Zhang, Leesa Lin, Hongjie Yu

**Affiliations:** 1 School of Public Health Fudan University Shanghai China; 2 Key Laboratory of Health Technology Assessment (National Health Commission) Fudan University Shanghai China; 3 Taub Institute for Research in Alzheimer’s Disease and the Aging Brain Columbia University New York, NY United States; 4 Department of Public Health Sciences College of Behavioral, Social and Health Sciences Clemson University Clemson, SC United States; 5 Department of Health Policy and Management College of Public Health University of Georgia Athens, GA United States; 6 Department of Infectious Disease Epidemiology London School of Hygiene & Tropical Medicine London United Kingdom; 7 Key Laboratory of Public Health Safety (Ministry of Education) Fudan University Shanghai China

**Keywords:** COVID-19, prevention, vaccination, behavior, children, China

## Abstract

**Background:**

The COVID-19 epidemic and the related containment strategies may affect parental and pediatric health behaviors.

**Objective:**

The goal of this study was to assess the change in children’s and adolescents’ prevention and vaccination behaviors amid China’s COVID-19 epidemic.

**Methods:**

We conducted a cross-sectional online survey in mid-March 2020 using proportional quota sampling in Wuhan (the epidemic epicenter) and Shanghai (a nonepicenter). Data were collected from 1655 parents with children aged 3 to 17 years. Children’s and adolescents’ prevention behaviors and regular vaccination behaviors before and during the epidemic were assessed. Descriptive analyses were used to investigate respondents’ characteristics, public health prevention behaviors, unproven protection behaviors, and vaccination behaviors before and during the COVID-19 epidemic. Univariate analyses were performed to compare differences in outcome measures between cities and family characteristics, using chi-square tests or Fisher exact tests (if expected frequency was <5) and analyses of variance. Multivariate logistic regressions were used to identify the factors and disparities associated with prevention and vaccination behaviors.

**Results:**

Parent-reported prevention behaviors increased among children and adolescents during the COVID-19 epidemic compared with those before the epidemic. During the epidemic, 82.2% (638/776) of children or adolescents always wore masks when going out compared with 31.5% (521/1655) before the epidemic; in addition, 25.0% (414/1655) and 79.8% (1321/1655) had increased their frequency and duration of handwashing, respectively, although only 46.9% (776/1655) went out during the epidemic. Meanwhile, 56.1% (928/1655) of the families took unproven remedies against COVID-19. Parent-reported vaccination behaviors showed mixed results, with 74.8% (468/626) delaying scheduled vaccinations and 80.9% (1339/1655) planning to have their children get the influenza vaccination after the epidemic. Regarding socioeconomic status, children and adolescents from larger families and whose parents had lower education levels were less likely to improve prevention behaviors but more likely to take unproven remedies. Girls were less likely than boys to always wear a mask when going out and wash their hands.

**Conclusions:**

Prevention behaviors and attitudes toward influenza vaccination have improved during the COVID-19 epidemic. Public health prevention measures should be continuously promoted, particularly among girls, parents with lower education levels, and larger families. Meanwhile, misinformation about COVID-19 remains a serious challenge and needs to be addressed by public health stakeholders.

## Introduction

On December 31, 2019, the government in Wuhan, China, announced an outbreak of a new infectious disease, formally named COVID-19. The COVID-19 outbreak spread quickly across China and the world [1]. In order to control the severe epidemic, the Chinese government launched a variety of containment strategies between January and April 2020, including lockdown policies, stay-at-home orders, school closures, and suspension of mass gatherings. The city of Wuhan, the epicenter of the COVID-19 outbreak, went through a complete lockdown from January 23 [2] to April 8, 2020 [3], whereas the Shanghai municipality, a city significantly affected by imported COVID-19 cases from Wuhan, activated the highest-level public health emergency response on January 24, 2020 [4], and later loosened it on March 24, 2020 [5]. Vaccination clinics were closed and then reopened in both Wuhan and Shanghai [6,7]. After the human-to-human transmission was confirmed on January 20, 2020, the National Health Commission of China issued the personal protection guidelines that included respiratory protection and hand hygiene [1], as well as health monitoring and social distancing [8]. Simultaneously, misinformation or rumors about COVID-19 started to spread on mass media and social media. The People’s Daily, the largest newspaper group in China, reported that Shuanghuanglian, an unproven herbal remedy, could inhibit COVID-19; 2 days later, it clarified that Shuanghuanglian cannot prevent COVID-19 [1]. The rumor that *garlic can prevent COVID-19* appeared on social media, and the People’s Daily refuted it again [1].

It is likely that the COVID-19 epidemic and subsequent containment strategies could have influenced the prevention and vaccination behaviors for adults and children [9]. Previous studies assessed the effectiveness of personal protective behaviors, indicating that behaviors such as mask-wearing, social distancing, and handwashing can help prevent COVID-19 transmission [10-12]. Some studies reported the positive impact of social distancing strategies, like the stay-at-home order, on the spread of the COVID-19 epidemic and health outcomes [13,14]. The public’s perception and compliance played an important role in the practice of personal protective behaviors, especially for children and adolescents who usually had a lower adherence to the recommended hygiene behaviors [15,16]. However, most studies focused on compliance with personal protective behaviors among adults during the COVID-19 epidemic [10,11,16-19], and few investigated children and adolescents. For Chinese adults, previous online surveys found that from February to March 2020, nearly 80% of adult residents complied with personal protection strategies, such as stay-at-home orders, mask-wearing, temperature self-monitoring, and hand sanitization, whereas adult migrant workers reported a higher compliance with mask-wearing in public places (95.7%) but a lower compliance with hand sanitization (70.9%) [20-22]. In addition, misinformation may bias the public’s perception and mislead the behavioral response to the epidemic [23]. It is plausible that these cognitive and behavioral challenges among the adult population could also occur among minors; therefore, assessing behavioral changes among children and adolescents may help identify the challenges associated with their adherence to containment strategies.

Public resources and services have long been unequally distributed among population subgroups in China and worldwide [24-28], which explained why the public responded to the COVID-19 epidemic differently in different places [29]. This epidemic may have led to various effects across population subgroups, and the public’s behavioral responses may differ by their demographic and socioeconomic status [30]. Previous studies have investigated the factors influencing adults’ behavioral responses to the COVID-19 epidemic and reported differential behaviors by socioeconomic status and gender [17,31-33]. Therefore, investigating the disparities in children’s behavioral responses and influencing factors would assist in identifying the subgroups with the highest needs, and could help in developing tailored interventions for children and adolescents to cope with the impacts of COVID-19. In addition, the COVID-19 epidemic and containment strategies were somehow different in the epicenter and nonepicenters, and comparison of prevention behaviors between the epicenter and nonepicenters could help us understand the influence of containment strategies with different degrees of severity.

Our study aimed to assess children’s and adolescents’ prevention behaviors and factors influencing their behavioral change amid the COVID-19 epidemic in China, and to further explore the disparities of behavioral changes among children and adolescents based on parent-reported records.

## Methods

### Study Design

From March 12 to 17, 2020, a cross-sectional online survey was conducted in Wuhan (the epicenter) and Shanghai (a nonepicenter) among parents with children aged 3 to 17 years. During our survey period, both Wuhan and Shanghai implemented the highest level of public health emergency response, including stay-at-home orders, closure of shops and schools, as well as suspension of mass gatherings [34]; as a result, the number of new COVID-19 cases reduced to 5 or lower in both cities. This study included children and adolescents at all stages of education, from kindergarten through high school, who were affected most by the school closure policy during the epidemic. During our survey period, children and adolescents spent most of their time staying at home with their parents, and all the personal prevention behaviors that we measured—going out, wearing of face masks, or handwashing—happened during the city lockdown. Although some parents resumed work in mid-February 2020 in Shanghai and in mid-March 2020 in Wuhan, most parents worked from home. Therefore, parents were children’s primary caregivers, and parents’ recall bias may not have been a big issue that affected the reliability of the measurement.

Our survey was operationalized by the ePanel data company [35], which maintains a national online survey panel with individuals’ phone numbers, email addresses, and basic information. This survey panel included millions of residents from major cities in China, including Shanghai and Wuhan. Residents in the two cities were randomly selected from this survey panel, and families with children aged 3 to 17 years were eligible to access the full survey. After accepting the survey invitation, one parent with children aged 3 to 17 years would provide informed consent and complete the questionnaire. In China, most households had a single child because of the one-child policy, especially in megacities such as Wuhan and Shanghai. Even if some households had multiple children, one parent was required to complete the questionnaire for only one of their children in our survey. The questionnaire (Multimedia Appendix 1) was pilot-tested with 30 participants who were excluded from this analysis. In addition, proportional quota sampling [36,37] was employed to ensure that respondents were demographically representative of the population according to local census data [38]. The final sample size—around 800 in each city—provided a sampling error of 3%. Once the numbers of survey respondents across children’s gender and age groups were reached in the same proportions of the Wuhan census and the Shanghai census, the predefined quota was then met and no more sampling was conducted. The study was approved by the Institutional Review Board (IRB) of the School of Public Health, Fudan University (IRB No. 2020-01-0801-S).

Between March 12 and 17, 2020, the survey was emailed to 73,000 residents in Wuhan and Shanghai, and 2960 (4.1%) residents accepted the survey invitation (Multimedia Appendix 2). In total, 2065 residents had children aged 3 to 17 years and were eligible to participate in the study, of whom 410 residents either did not finish or had missing data in their responses, leaving data from 1655 respondents (816 in Wuhan [49.3%] and 839 in Shanghai [50.7%]) in the final analytic sample.

### Measures

This study classified the behaviors of interest among children or adolescents into three categories: (1) public health prevention behaviors (ie, mask-wearing, handwashing, self-monitoring of COVID-19 symptoms, and social distancing), (2) unproven protection behaviors (ie, use of unproven or potentially harmful treatments), and (3) vaccination behaviors (ie, influenza vaccination and regularly scheduled vaccinations, but not COVID-19 vaccination). These behaviors were measured twice: once during the COVID-19 epidemic and once before the epidemic. Prevention behaviors were measured by the frequency of children or adolescents wearing masks when going out before and during the epidemic, the frequency and duration of washing hands after coming home before and during the epidemic, and the frequency of monitoring body temperature and going out during the epidemic. This study used the monitoring of body temperature to represent the behavior of self-monitoring of COVID-19 symptoms, since fever is one of the main symptoms of COVID-19. Unproven protection behaviors were measured by the instances of buying or taking unproven herbal remedies or garlic during the epidemic, which were believed to prevent COVID-19 through rumors. Vaccination behaviors included the delay of scheduled vaccinations, whether the parent was informed about alternative vaccination arrangements during the epidemic, and the receipt of the influenza vaccination in the past and their future intentions after the epidemic.

Data about the characteristics of parents and children or adolescents were also collected, including parents’ education level, household size (ie, number of family members living together), children’s or adolescents’ gender and age, and whether there were confirmed or suspected COVID-19 cases in their neighborhood. The questionnaire is included in Multimedia Appendix 1.

### Statistical Analysis

Descriptive analysis was used to investigate respondents’ characteristics, public health prevention behaviors, unproven protection behaviors, and vaccination behaviors before and during the COVID-19 epidemic. Univariate analyses were performed to compare differences in outcome measures between cities and family characteristics, using chi-square tests or Fisher exact tests (if expected frequency was <5) for categorical measures and analyses of variance for continuous measures. Multivariate logistic regressions were used to identify the factors and disparities associated with prevention and vaccination behaviors during the epidemic. Each of the following eight behavioral indicators during the epidemic was modeled as a dependent variable separately: (1) frequency of mask-wearing (always vs not always), (2) frequency of handwashing (always vs not always), (3) duration of hand washing (<40 seconds vs ≥40 seconds), (4) frequency of monitoring body temperature (>3 times/week vs ≤3 times/week), (5) whether they went outside or not, (6) whether they took unproven remedies or not, (7) whether they delayed scheduled vaccinations or not, and (8) whether they planned to receive the influenza vaccination or not after the epidemic. Independent variables in each regression model included all available characteristics of the parents and children or adolescents. The proportions and odds ratios (ORs) with 95% CIs were reported. All statistical analyses were performed using Stata 14.0 (StataCorp LP).

## Results

### Characteristics of Respondents

The sample characteristics are presented in Table 1. The gender and age distributions of sampled children and adolescents were similar to the two cities’ censuses, indicating the representativeness of our sample. Of 1655 respondents, 1077 (65.1%) were mothers of children and 1225 (74.0%) reported obtaining bachelor’s degrees or above; in addition, there were an average of 3.5 (SD 1.30) members in the sampled families. There were no significant differences in respondents’ characteristics between Wuhan and Shanghai. Due to the more severe epidemic in Wuhan, more respondents in Wuhan (277/816, 33.9%) reported that there were confirmed or suspected COVID-19 cases in their neighborhood, compared to those in Shanghai (88/839, 10.5%).

**Table 1:** Characteristics of respondents in Wuhan and Shanghai during the COVID-19 epidemic, March 2020.

Characteristic		Total (N=1655)	Wuhan (n=816)	Shanghai (n=839)	*P* value	
**Gender of child or adolescent, n (%)**						.75^a^
	Male	830 (50.2)	406 (49.8)	424 (50.5)		
	Female	825 (49.9)	410 (50.3)	415 (49.5)		
**Age of child or adolescent (years), n (%)**						.84^a^
	3-5	321 (19.4)	160 (19.6)	161 (19.2)		
	6-9	432 (26.1)	217 (26.6)	215 (25.6)		
	10-14	359 (21.7)	180 (22.1)	179 (21.3)		
	15-17	543 (32.8)	259 (31.7)	284 (33.9)		
**Respondent** **, n (%)**						.10^a^
	Mother of child or adolescent	1077 (65.1)	547 (67.0)	530 (63.2)		
	Father of child or adolescent	578 (34.9)	269 (33.0)	309 (36.8)		
**Education level of parent** **, n (%)**						.32^a^
	High school or below	103 (6.2)	45 (5.5)	58 (6.9)		
	Some college	327 (19.8)	170 (20.8)	157 (18.7)		
	Bachelor’s degree or above	1225 (74.0)	601 (73.7)	624 (74.4)		
Household size, mean (SD)		3.5 (1.30)	3.5 (1.26)	3.5 (1.34)	.39^b^	
**COVID-19 cases in neighborhood** **, n (%)**						<.001^a^
	Yes	365 (22.1)	277 (33.9)	88 (10.5)		
	No or unclear	1290 (78.0)	539 (66.1)	751 (89.5)		

^a^*P* value was calculated from a chi-square test.

^b^*P* value was calculated from an analysis of variance.

### Prevention and Vaccination Behaviors Among Children and Adolescents During the Epidemic

**Figure 1:**
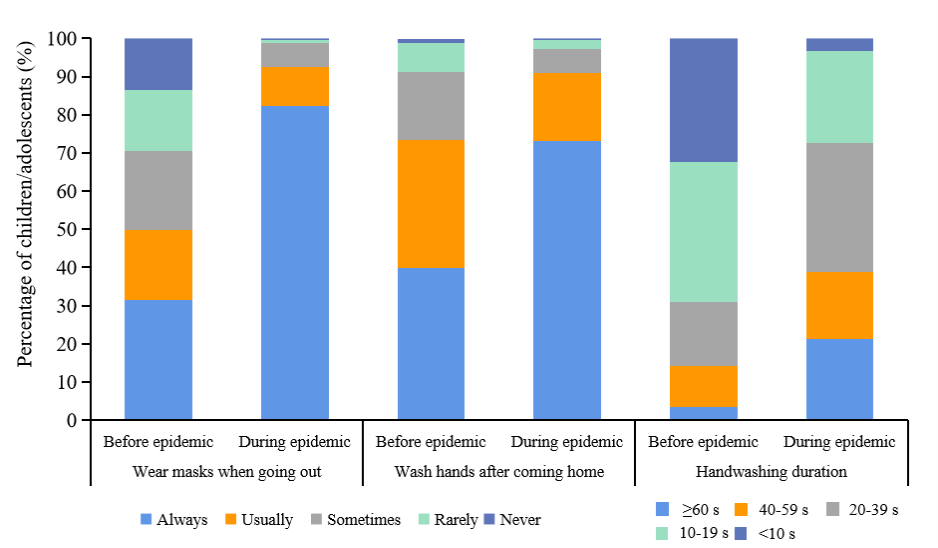
Comparison of prevention behaviors among children and adolescents in Wuhan and Shanghai before and during the COVID-19 epidemic, March 2020.

Figure 1 and Table 2 describe prevention behaviors, unproven protection measures, and vaccination behaviors among children and adolescents before and during the COVID-19 epidemic. Only 46.9% (776/1655) of children or adolescents went out since the beginning of the epidemic (Table 2). There were 82.2% (638/776) of children or adolescents who always wore masks when going out during the epidemic compared with 31.5% (521/1655) before the epidemic (Figure 1). Primary reasons for not always wearing masks during the epidemic included having no masks (61/144, 42.4%), children and adolescents thinking mask-wearing was unattractive or uncomfortable (41/144, 28.5%), and parents thinking mask-wearing had limited protective effect (37/144, 25.7%) (Table 2). During the epidemic, both the frequency and duration of handwashing after coming home increased significantly among children or adolescents (Figure 1), with 25.0% (414/1655) and 79.8% (1321/1655) having increased the frequency and duration of handwashing, respectively. During the epidemic, 57.5% (952/1655) of families monitored children’s or adolescents’ body temperature more than 3 times each week (Table 2). There were 56.1% (928/1655) of families who bought or took unproven herbal remedies, and 30.3% (501/1655) took garlic, as a result of a rumor that it was protective (Table 2).

Among 626 children or adolescents with scheduled vaccinations, 468 (74.8%) delayed vaccination, of whom 70.1% (328/468) delayed more than 2 weeks and 55.6% (260/468) were worried about vaccination delay (Table 2). A total of 90.9% (569/626) of parents reported being informed about alternative vaccination arrangements during the epidemic. During the 2019 flu season, 54.7% (905/1655) of families had taken their children or adolescents to receive the influenza vaccination, and 80.9% (1339/1655) of parents intended to vaccinate their children or adolescents against influenza in the future after the epidemic, a rate much higher than that before the epidemic (Table 2).

Findings of univariate analysis between Wuhan and Shanghai are shown in Table 2 and by respondents’ characteristics in Multimedia Appendix 3. In comparison with the Shanghai sample, parents in Wuhan were significantly more likely to monitor body temperature of their children or adolescents, and their children were less likely to go out during the epidemic. There was no significant difference in taking unproven remedies or garlic to prevent COVID-19 between the two cities. More children and adolescents in Wuhan had delayed their scheduled vaccinations and delayed for a longer time than those in Shanghai, but a similar proportion of parents in the two cities expressed concerns about the delay and were informed of alternative vaccination arrangements. In addition, there were no significant differences in parents’ future intentions to vaccinate their children against influenza after the epidemic between the two cities.

**Table 2:** Prevention, unproven protection, and vaccination behaviors among children and adolescents in Wuhan and Shanghai during the COVID-19 epidemic, March 2020.

Prevention, unproven protection, and vaccination behavior				Total (N=1655), n (%)		Wuhan (n=816), n (%)		Shanghai (n=839), n (%)		*P* value	
**Public health prevention behavior**											
	**Frequency of child or adolescent going out during the epidemic**										<.001^a^
		Never	879 (53.1)		479 (58.7)		400 (47.7)				
		<1 time/week	505 (30.5)		229 (28.1)		276 (32.9)				
		1-2 times/week	220 (13.3)		93 (11.4)		127 (15.1)				
		3-5 times/week	47 (2.8)		13 (1.6)		34 (4.1)				
		Nearly everyday	4 (0.2)		2 (0.3)		2 (0.2)				
	**Reasons for child or adolescent not always wearing a mask when going out during the epidemic (n=144)^b^**										.64^a^
		They had no masks	61 (42.4)		21 (39.6)		40 (44.0)				
		Child or adolescent thought mask-wearing was unattractive	26 (18.1)		10 (18.9)		16 (17.6)				
		Child or adolescent thought mask-wearing was uncomfortable	15 (10.4)		4 (7.6)		11 (12.1)				
		Parent thought masks had limited protective effects	37 (25.7)		17 (32.1)		20 (22.0)				
		Parent thought the epidemic was not severe	5 (3.5)		1 (1.9)		4 (4.4)				
	**Frequency of monitoring body temperature of child or adolescent during the epidemic**										<.001^c^
		Never	120 (7.3)		27 (3.3)		93 (11.1)				
		≤1 time/week	264 (16.0)		106 (13.0)		158 (18.8)				
		2-3 times/week	319 (19.3)		125 (15.3)		194 (23.1)				
		4-5 times/week	366 (22.1)		209 (25.6)		157 (18.7)				
		6-7 times/week	586 (35.4)		349 (42.8)		237 (28.3)				
**Unproven protection behavior**											
	Family members bought or took unproven herbal remedies to prevent COVID-19			928 (56.1)		451 (55.3)		477 (56.9)		.52^c^	
	Family members took unproven garlic remedy to prevent COVID-19			501 (30.3)		252 (30.9)		249 (29.7)		.59^c^	
**Vaccination behavior**											
	Delayed the scheduled vaccinations for child or adolescent during the epidemic (n=626)^b^			468 (74.8)		239 (78.6)		229 (71.1)		.03^c^	
	**How long child or adolescent vaccination was delayed during the epidemic (n=468)^b^**										.03^c^
		<2 weeks	140 (30.0)		68 (28.5)		72 (31.4)				
		2 weeks to 1 month	143 (30.6)		86 (36.0)		57 (24.9)				
		>1 month	185 (39.5)		85 (35.6)		100 (43.7)				
	Parent was worried about the delay of child or adolescent vaccination (n=468)^b^			260 (55.6)		140 (58.6)		120 (52.4)		.18^c^	
	Parent was informed of alternative vaccination arrangements during the epidemic (n=626)^b^			569 (90.9)		282 (92.8)		287 (89.1)		.11^c^	
	Child or adolescent had received influenza vaccination in 2019 flu season			905 (54.7)		467 (57.2)		438 (52.2)		.04^c^	
	Planned to vaccinate child or adolescent against influenza after the epidemic			1339 (80.9)		655 (80.3)		684 (81.5)		.52^c^	

^a^*P* value was calculated from a Fisher exact test.

^b^These questions were only asked based on the response to a prior question, and the total number of respondents was marked separately.

^c^*P* value was calculated from a chi-square test.

### Factors Associated With Prevention and Vaccination Behaviors Among Children and Adolescents During the Epidemic

Table 3 presents the factors associated with children’s prevention and vaccination behaviors during the epidemic using multivariate logistic regressions. This analysis showed that during the epidemic, children or adolescents in Shanghai, compared to Wuhan, were more likely to go outside (OR 1.56, 95% CI 1.26-1.92), less likely to monitor body temperature more than 3 times per week (OR 0.42, 95% CI 0.34-0.52), and less likely to delay scheduled vaccinations (OR 0.60, 95% CI 0.40-0.88). The education level of parents was significantly associated with prevention, unproven protection, and vaccination behaviors, with negative associations with the prevalence of going outside and taking unproven remedies and positive associations with other behaviors. Specifically, children and adolescents whose parents had lower education levels were more likely to go outside and take unproven remedies, and they were less likely to wear masks, wash hands, and monitor body temperature during the epidemic. Their parents were also less likely to delay scheduled vaccinations during the epidemic and had lower intentions of having their children receive the influenza vaccination after the epidemic, compared to children and adolescents whose parents had higher levels of education. During the epidemic, children and adolescents from larger families were more likely to go outside and take unproven remedies as well, while they were less likely to wash their hands for 40 seconds or more or take their body temperature more than 3 times per week. Girls were less likely to wear masks (OR 0.61, 95% CI 0.41-0.91) or wash their hands (OR 0.64, 95% CI 0.45-0.90) than boys during the epidemic. Children’s and adolescents’ ages were significantly and negatively associated with the taking of unproven remedies and intentions to receive the influenza vaccination after the epidemic. Having a father or mother as their caregiver and COVID-19 prevalence in their neighborhood also influenced some preventive behaviors of children and adolescents. 

**Table 3:** Factors associated with prevention, unproven protection, and vaccination behaviors among respondents in Wuhan and Shanghai during the COVID-19 epidemic by multivariate logistic regression, March 2020.

Characteristic		Public health prevention behavior, OR^a^ (95% CI)						Unproven protection behavior, OR (95% CI)		Vaccination behavior, OR (95% CI)			
		Always wear a mask (n=776)	Always wash hands (n=774)	Wash hands ≥40 seconds (N=1655)	Monitor body temperature >3 times/week (N=1655)	Go outside (N=1655)	Take unproven remedies (N=1655)		Delay scheduled vaccination (n=626)			Plan influenza vaccination after epidemic (N=1655)	
Shanghai (ref^b^: Wuhan)		0.69 (0.46-1.04)	1.09 (0.77-1.55)	0.84 (0.68-1.04)	0.42** (0.34-0.52)	1.56** (1.26-1.92)	1.16 (0.94-1.43)		0.60** (0.40-0.88)			1.22 (0.93-1.59)	
**Education of parent (ref:** **bachelor’s degree or above)**													
	Some college	0.42** (0.27-0.64)	0.37** (0.26-0.55)	0.75* (0.58-0.98)	0.55** (0.42-0.71)	1.46** (1.13-1.88)	1.30* (1.01-1.69)				0.75 (0.46-1.21)		0.51** (0.37-0.69)
	High school or below	0.30** (0.17-0.55)	0.19** (0.11-0.33)	0.93 (0.60-1.42)	0.47** (0.30-0.71)	1.66* (1.08-2.53)	1.12 (0.73-1.70)				0.30** (0.15-0.59)		0.63 (0.38-1.05)
Household size		0.96 (0.87-1.06)	1.01 (0.91-1.11)	0.89* (0.80-0.99)	0.83** (0.75-0.93)	1.27** (1.13-1.43)	1.19** (1.06-1.34)				1.10 (0.96-1.26)		1.02 (0.92-1.13)
Female child or adolescent (ref: male)		0.61* (0.41-0.91)	0.64* (0.45-0.90)	1.23 (0.99-1.51)	1.22 (0.99-1.51)	1.01 (0.82-1.24)	0.96 (0.78-1.18)				1.10 (0.75-1.63)		0.79 (0.61-1.04)
**Age of child or adolescent (years) (ref: 3-5 years)**													
	6-9	1.02 (0.60-1.74)	1.61 (0.98-2.67)	0.56** (0.41-0.76)	0.89 (0.65-1.21)	1.12 (0.83-1.51)	0.71* (0.52-0.97)				1.90** (1.19-3.05)		0.87 (0.55-1.37)
	10-14	1.21 (0.65-2.24)	1.37 (0.79-2.37)	1.00 (0.74-1.37)	0.72* (0.52-0.99)	0.81 (0.59-1.12)	0.63** (0.46-0.87)				1.92* (1.04-3.55)		0.56* (0.36-0.87)
	15-17	1.65 (0.96-2.82)	1.15 (0.72-1.82)	1.10 (0.83-1.46)	1.03 (0.77-1.38)	1.28 (0.96-1.70)	0.49** (0.37-0.66)				1.32 (0.80-2.19)		0.33** (0.22-0.50)
Father respondent (ref: mother)		0.92 (0.61-1.37)	0.78 (0.55-1.12)	1.40** (1.12-1.74)	0.75* (0.60-0.94)	1.92** (1.55-2.39)	1.19 (0.96-1.48)		1.15 (0.76-1.75)			0.56** (0.43-0.73)	
COVID-19 cases in neighborhood		1.27 (0.77-2.10)	2.16** (1.35-3.44)	1.22 (0.95-1.57)	1.19 (0.92-1.55)	1.09 (0.84-1.40)	1.46** (1.13-1.89)		0.74 (0.48-1.15)			1.31 (0.94-1.83)	
Received influenza vaccination in 2019 season		N/A^c^	N/A	N/A	N/A	N/A	N/A		N/A			2.05** (1.57-2.67)	

^a^OR: odds ratio.

^b^ref: reference.

^c^N/A: not applicable; this item was only applicable to the vaccination behavior *plan influenza vaccination after epidemic*.

*P<.05.

**P<.01.

## Discussion

### Principal Findings

This study provides evidence regarding the change of public health prevention behaviors, unproven protection behaviors, and vaccination behaviors among children and adolescents amid the COVID-19 epidemic in China. In this online survey of 1655 parents with children aged 3 to 17 years in Wuhan and Shanghai, there was an increase in the frequency of prevention behaviors and unproven protection behaviors, as compared with the pre-epidemic time. We documented the parent-reported delay of scheduled vaccinations and their intention to receive the influenza vaccination after the epidemic. This study also observed disparities in prevention behaviors, unproven remedies taken, and vaccination behaviors by child gender, parental education attainment, and family size.

This study found prevention behavior changes among children and adolescents (ie, increasing frequencies of mask-wearing and self-monitoring of COVID-19 symptoms, an increasing frequency and duration of handwashing, and a decrease in the frequency of going outside during the COVID-19 epidemic). The increasing frequency of washing hands and mask use was consistent with previous studies in Europe and North America [17,39]. On the other hand, few previous studies compared the prevalence of mask use, symptom self-monitoring, and going outside during and before the epidemic. The mask-wearing mandate has been proven to be associated with mitigating the spread of COVID-19 [10,11]. Previous studies also provided evidence that the shutdown policy was associated with delaying the COVID-19 epidemic in other cities and with reductions in total incidence [34,40]. Thus, it is of great importance to maintain the recommendation of prevention behaviors, which will warrant maintenance of the existing strategies to protect children and adolescents from the transmission of COVID-19. It is worth noting that this study was conducted in mid-March 2020, when China was going through a period of critical shortages of personal protective equipment; thus, the observed insufficient protection for children and adolescents may be closely related to the limited supply of personal protective equipment. Additionally, for the implementation of the mandate, we need to be aware that children’s adherence is usually lower than that of adults, and their willingness should be taken into consideration, specifically [15]. As reported in this study, besides the primary reason of lack of access to masks, the other reason for children and adolescents’ lack of compliance with the mask mandate could be their reluctance. Thus, it is necessary to obtain children’s cooperation with the help of their parents and to adopt other measures simultaneously, such as staying at home and keeping social distance, if children’s compliance with the mask mandate is too difficult to achieve [15].

Our findings about unproven protection behaviors showed that over half of the families bought or took unproven herbal remedies. Since disease incidence and mortality are increasing globally during the COVID-19 epidemic, using unproven remedies is an understandable temptation [41]. Yet, misinformation related to these therapies has spread online at a surprising rate during the epidemic, which has a negative impact on controlling the transmission of COVID-19 [20] and could ultimately result in poor health outcomes among individuals [42]. Thus, to detect and debunk misinformation or anecdotal information, multisectoral efforts are needed from public health stakeholders, such as social media, health care professionals, or experts. Decision makers should also help regulate relevant law enforcement to ensure that accurate information on COVID-19 is provided.

This study also found a delay in the use of vaccines during the epidemic but an increase in the demand for influenza vaccination, which might be an indirect consequence of the shutdown policy. All vaccinations were expected to be delayed due to the closure of vaccination clinics and risk of COVID-19 infection, regardless of whether they were covered by the National Immunization Program in China, a program that aims to provide free immunization services for Chinese children. A notable decline in childhood vaccination was also observed in the United Kingdom, Ireland, the United States, and globally [43-45]. Delaying vaccination appears to signal that there may be a spike in demand for vaccines immediately after the COVID-19 epidemic [12]. Parents may still worry about the risk of getting COVID-19 when going out or in a crowded vaccination clinic, and are hesitant to get their children vaccinated. In addition, COVID-19 also increased people’s attention toward influenza vaccination, and, as reported in the Results section, 80.9% of parents planned to have their child receive the influenza vaccination after the epidemic. In China, where the influenza vaccination rate has been low—9.4% among the general population—compared with other countries [46], this increase in demand for influenza vaccinations could be one of the unexpected beneficial consequences of the COVID-19 epidemic. This means that, at least in Shanghai and Wuhan, there will be a dramatic increase in the utilization of influenza vaccination, which reminds the vaccination providers to prepare for and store more vaccine doses. Since September 2020, China has seen a great demand for, and a shortage of, influenza vaccination. In the long run, since the increase in demand for vaccinations may be sustained beyond the COVID-19 epidemic, the government should develop a sustainable and effective plan for pediatric vaccine schedules.

The disparities in prevention and vaccination behaviors existed among children and adolescents by child gender, parental education attainment, and family size. Girls wore masks and washed hands at significantly lower frequencies than boys during the epidemic, but no significant differences by gender were found for other prevention behaviors. The children and adolescents who had parents with lower education levels and who came from larger families were more likely to have used unproven remedies and less likely to have exhibited the recommended prevention behaviors. First, it is worth noting that the findings of less frequent mask-wearing and handwashing among girls were opposite to the findings among adults, wherein female adults wore masks and washed hands at higher frequencies [16,18,19]. Girls at those ages might have concerns about how they are looked at by others and their body size, shape, or weight [47,48]. Hence, the low frequency of mask-wearing among girls may be due to their concerns about appearance. Second, the findings regarding behavior disparities as a function of parental education attainment were consistent with previous studies, which explained this result as an increased awareness of perceived susceptibility and severity of disease [33]. Parents with higher educational attainment may have a better understanding of the effectiveness, perception, and guidance of public health prevention behaviors [49]. Third, the behavior disparity regarding unproven remedy use as a function of household size may have resulted from a higher risk of exposure to misinformation, as people living in larger households could experience a higher probability of exposure to misinformation and spread it to other family members. In addition, given that the average number of family members in the recruited families in this study was 3.5, our study sample may include children who live with grandparents. Since the elderly have been more inclined to share misinformation compared to younger adults [50], larger families with grandparents have been more likely to take unproven remedies. Thus, more attention is needed regarding health education for girls under the context of an epidemic, and public health stakeholders should tailor expanded multisectoral efforts to children and adolescents living in larger families whose parents have lower educational levels.

### Limitations

This study is subject to several limitations. First, the results may be affected by selection bias from an online survey. While almost all families have access to the internet or a telephone in Wuhan and Shanghai [51], and quota sampling enhanced the representativeness of our sample to minimize selection bias, we are aware that our sample is not a probabilistic random sample of residents from the two cities. Similar to other online surveys [52], the response rate was below 10% in our study. Second, there may be recall bias due to the self-reported data. Since we conducted the survey while most children and adolescents were still staying at home with their parents, recall bias might be limited as we were measuring ongoing behavioral patterns. Meanwhile, while the anonymous survey addressed the concern of reporting bias regarding sensitive information, social desirability bias could still exist to affect the reporting of behaviors that have been mandated by the government. Finally, since our survey took place in mid-March 2020, the results of this study may be applicable to the early phase of the COVID-19 outbreak. As the COVID-19 pandemic was initially under control in China at that time, this study may have limited significance for current prevention. In addition, the behaviors performed and measures taken by parents were not reflected in this paper. As the epidemic evolves, we plan to conduct follow-up surveys to study the long-term behavioral changes among children and their parents. A better understanding of how the epidemic affects the behaviors of children and their parents can help guide future prevention strategies.

### Conclusions

During the COVID-19 epidemic, children and adolescents improved in their prevention behaviors and attitudes toward influenza vaccination. Public health prevention measures should be continuously promoted, particularly among girls, parents with lower educational attainment, and larger families. Misinformation about COVID-19 remains a serious challenge and needs to be addressed by public health stakeholders. In addition, the epidemic led to a serious delay of regular vaccination services yet increased the willingness to get the influenza vaccination; thus, it is vital to ensure a sufficient supply of different kinds of vaccines to meet the surging vaccination need after the pandemic [53].

## Appendix

Multimedia Appendix 1 Study questionnaire.

Multimedia Appendix 2 Flowchart of participant recruitment.

Multimedia Appendix 3 Prevention and vaccination behaviors among children and adolescents by respondents’ characteristics during the COVID-19 epidemic, March 2020.

## Abbreviations

IRB: Institutional Review Board
OR: odds ratio
