# Supplementary material for: The Influence of the COVID-19 Epidemic on Prevention and Vaccination Behaviors Among Chinese Children and Adolescents: Cross-sectional Online Survey Study
Source: JMIR Public Health Surveill. 2021 May 26;7(5):e26372. doi: 10.2196/26372 (PMC8158530; doi:10.2196/26372)
Supplement: Multimedia Appendix 1 [file publichealth_v7i5e26372_app1.docx]

**Multimedia Appendix 1: Study questionnaire**

**Prevention and Vaccination Behaviors among Chinese Children and Adolescents before and during COVID-19 epidemic**

1. **Characteristics**
2. Where do you live currently?

①Wuhan ②Shanghai ③None of the above (End the survey)

1. The gender of child/adolescent?

①Male ②Female

1. The age of child/adolescent? (years old)

①3-5 ②6-9 ③10-14 ④15-17 ⑤None of the above (End the survey)

1. How many people in your family live together (more than 4 days a week)?
2. What is your education level?

①Primary School or Below ②Junior high school ③Senior high school or technical secondary school ④Junior college ⑤Undergraduate college ⑥Postgraduate or above

1. Are there any confirmed or suspected COVID-19 cases in your neighborhood?

①Yes ②No ③Not clear

1. What’s your relationship with child/adolescent?

①Mother ②Father ③Other (End the survey)

1. **Public health prevention behaviors**
2. Before the COVID-19 epidemic (before December 31, 2019), how often did your child/adolescent wear masks when going out?

①Always ②Usual ③Sometimes ④Rare ⑤Never

1. How often does your child/adolescent go out during the COVID-19 epidemic?

①Never (Skip to 12) ②<1/week ③1-2/week ④3-5/week ⑤Nearly everyday

1. During the COVID-19 epidemic, how often does your child/adolescent wear masks when going out?

①Always (Skip to 12) ②Usual ③Sometimes ④Rare ⑤Never

1. During the COVID-19 epidemic, why does your child/adolescent not always wear masks when going out?

①They had no masks

②Child/adolescent thought mask-wearing was unattractive

③Child/adolescent thought mask-wearing was uncomfortable

④Parent thought masks had limited protective effect

⑤Parent thought the epidemic was not severe

1. Before the COVID-19 epidemic (before December 31, 2019), how often did your child/adolescent wash hands immediately after coming home?

①Always ②Usual ③Sometimes ④Rare ⑤Never

1. During the COVID-19 epidemic, how often does your child/adolescent wash hands immediately after coming home?

①Always ②Usual ③Sometimes ④Rare ⑤Never ⑥Never go out

1. Before the COVID-19 epidemic (before December 31, 2019), how long did your child/adolescent wash hands each time?

①<10s ②10-19s ③20-39s ④40-59s ⑤≥60s

1. During the COVID-19 epidemic, how long did your child/adolescent wash hands each time?

①<10s ②10-19s ③20-39s ④40-59s ⑤≥60s

1. During the COVID-19 epidemic, how often do you monitor body temperature for your child/adolescent?

①Never ②≤1/week ③2-3/week ④4-5/week ⑤6-7/week

1. **Unproven protection behaviors**
2. During the COVID-19 epidemic, have you or your family members bought or taken unproven herbal remedies to prevent COVID-19?

①Have bought but not taken ②Have bought and taken ③Have taken unproven herbal remedies bought before ④Have not bought or taken

1. During the COVID-19 epidemic, have you or your family members taken garlic to prevent COVID-19?

①Yes ②No

1. **Vaccination behaviors**
2. During the COVID-19 epidemic, does your child/adolescent have scheduled vaccinations?

①Yes ②No

1. During the COVID-19 epidemic, have you delayed scheduled vaccines for your child/adolescent?

①Yes ②No

1. During the COVID-19 epidemic, how long was your child/adolescent vaccination delayed?

①<2 weeks ②2 weeks - 1 month ③>1 month

1. During the COVID-19 epidemic, are you worried about the delay of child/adolescent vaccination?

①Very worried ②Worried ③Neutral ④Not so worried ⑤Not worried at all

1. During the COVID-19 epidemic, are you informed alternative vaccination arrangement?

①Yes ②No

1. Has your child/adolescent received influenza vaccination in 2019 flu season?

①Yes ②No

1. Do you plan to vaccinate child/adolescent against influenza after the epidemic?
2. Yes ②No
