# Supplementary figures and images for: The Influence of the COVID-19 Epidemic on Prevention and Vaccination Behaviors Among Chinese Children and Adolescents: Cross-sectional Online Survey Study
Source: JMIR Public Health Surveill. 2021 May 26;7(5):e26372. doi: 10.2196/26372 (PMC8158530; doi:10.2196/26372)

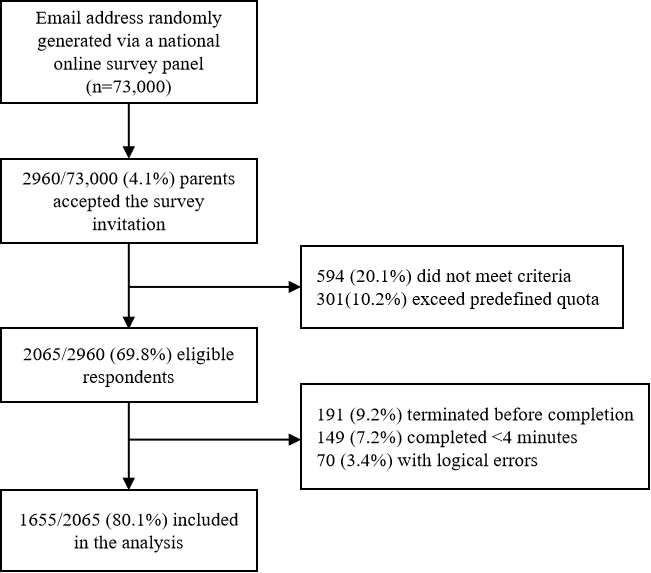

Supplement: Multimedia Appendix 2 [file publichealth_v7i5e26372_app2.png]
