# Supplementary material for: The Influence of the COVID-19 Epidemic on Prevention and Vaccination Behaviors Among Chinese Children and Adolescents: Cross-sectional Online Survey Study
Source: JMIR Public Health Surveill. 2021 May 26;7(5):e26372. doi: 10.2196/26372 (PMC8158530; doi:10.2196/26372)
Supplement: Multimedia Appendix 3 [file publichealth_v7i5e26372_app3.docx]

**Multimedia Appendix 3:** **Prevention and vaccination behaviors (%) among children and adolescents by respondents’ characteristics during the COVID-19 epidemic, March 2020**

|  | Public health prevention behaviors | | | | | Unproven protection behaviors | Vaccination behaviors | |
| --- | --- | --- | --- | --- | --- | --- | --- | --- |
|  | Always wear masks | Always wash hands | Wash hands ≥40s | Monitor body temperature > 3/week | Go outside | Take unproven remedies | Delay of scheduled vaccination | Plan influenza vaccination after epidemic |
| City |  |  |  |  |  |  |  |  |
| Wuhan | 85.2 | 72.8 | 41.1 | 68.4 | 41.3 | 55.3 | 78.6 | 80.3 |
| Shanghai | 80.0 | 73.1 | 36.5 | 47.0 | 52.3 | 56.9 | 71.1 | 81.5 |
| P value | 0.060 | 0.930 | 0.056 | <0.001 | <0.001 | 0.516 | 0.031 | 0.516 |
| Education of parent |  |  |  |  |  |  |  |  |
| Bachelor or above | 87.2 | 80.2 | 40.4 | 61.3 | 43.9 | 54.2 | 77.3 | 83.6 |
| Some college | 73.0 | 61.2 | 33.0 | 48.0 | 54.4 | 62.1 | 72.6 | 72.2 |
| High school or below | 65.0 | 43.3 | 36.9 | 42.7 | 58.3 | 59.2 | 51.2 | 76.7 |
| P value | <0.001 | <0.001 | 0.048 | <0.001 | <0.001 | 0.031 | 0.001 | <0.001 |
| Household size |  |  |  |  |  |  |  |  |
| ≤3 people | 84.0 | 74.1 | 41.7 | 61.0 | 43.6 | 52.1 | 74.8 | 80.9 |
| ≥4 people | 78.6 | 70.7 | 31.0 | 48.5 | 55.4 | 66.4 | 74.8 | 81.0 |
| P value | 0.064 | 0.312 | <0.001 | <0.001 | <0.001 | <0.001 | 0.995 | 0.934 |
| Gender of child/adolescent |  |  |  |  |  |  |  |  |
| Male | 85.9 | 77.4 | 37.4 | 54.6 | 48.7 | 57.0 | 74.7 | 81.7 |
| Female | 78.2 | 68.3 | 40.1 | 60.5 | 45.1 | 55.2 | 74.8 | 80.1 |
| P value | 0.005 | 0.004 | 0.247 | 0.015 | 0.144 | 0.452 | 0.966 | 0.418 |
| Age of child/adolescent |  |  |  |  |  |  |  |  |
| 3-5 years | 78.4 | 68.0 | 41.4 | 58.3 | 47.7 | 67.3 | 66.9 | 88.5 |
| 6-9 years | 79.1 | 77.7 | 28.2 | 56.9 | 47.7 | 58.3 | 79.0 | 87.5 |
| 10-14 years | 82.5 | 74.8 | 41.8 | 54.3 | 38.2 | 53.8 | 80.4 | 82.2 |
| 15-17 years | 86.4 | 71.4 | 43.5 | 59.7 | 51.6 | 49.2 | 74.5 | 70.4 |
| P value | 0.100 | 0.186 | <0.001 | 0.447 | 0.001 | <0.001 | 0.025 | <0.001 |
| Respondents |  |  |  |  |  |  |  |  |
| Father | 83.4 | 72.4 | 43.4 | 52.6 | 57.3 | 59.2 | 74.6 | 76.3 |
| Mother | 81.4 | 73.4 | 36.2 | 60.2 | 41.3 | 54.4 | 74.8 | 83.4 |
| P value | 0.463 | 0.757 | 0.004 | 0.003 | <0.001 | 0.063 | 0.960 | <0.001 |
| COVID-19 cases in neighborhood |  |  |  |  |  |  |  |  |
| Yes | 84.6 | 82.1 | 42.7 | 65.2 | 46.3 | 63.3 | 71.1 | 82.5 |
| No or unclear | 81.6 | 70.5 | 37.6 | 55.4 | 47.1 | 54.0 | 76.0 | 80.5 |
| P value | 0.356 | 0.003 | 0.075 | 0.001 | 0.799 | 0.002 | 0.227 | 0.391 |
| N | 776 | 774 | 1655 | 1655 | 1655 | 1655 | 626 | 1655 |
